# A Digital Coaching Intervention for Cancer Survivors With Job Loss: Retrospective Study

**DOI:** 10.2196/31966

**Published:** 2021-11-23

**Authors:** Jonathon Lo, Kieran Ballurkar, Simonie Fox, Kate Tynan, Nghiep Luu, Michael Boyer, Raghav Murali-Ganesh

**Affiliations:** 1 Faculty of Medicine University of Melbourne Melbourne Australia; 2 CancerAid Sydney Australia; 3 AIA Australia Sydney Australia; 4 Department of Oncology Chris O’Brien Lifehouse Sydney Australia; 5 Faculty of Medicine University of Sydney Sydney Australia; 6 Northern Sydney Cancer Centre Royal North Shore Hospital Sydney Australia

**Keywords:** cancer survivors, employment, absenteeism, mobile app, software, return to work

## Abstract

**Background:**

Returning to work is a key unmet need for working-age cancer survivors.

**Objective:**

This study sought to evaluate return-to-work outcomes of a multidisciplinary intervention provided as routine employee support.

**Methods:**

In a retrospective cohort analysis, patients with cancer and more than 3 months of absence from work were provided with an intervention consisting of digital resources and calls with a health coach. Propensity score matching was used to define a similar cohort of cancer patients absent from work, who were not offered the coaching intervention. The return-to-work rate as a percentage of all participants and secondary outcomes, such as the rate of death, were measured. The median time to return to work was compared between the cohorts using the Kaplan-Meier method.

**Results:**

A total of 220 participants were enrolled in the intervention, of which 125 met the criteria for analysis. The median follow-up from cancer diagnosis was 79 weeks (IQR 60-106 weeks). In the matched control group, 22 (17.6%) participants returned to work compared with 38 (30.4%) in the intervention group (*P*=.02). Additionally, 19 (15.2%) matched controls died prior to claim closure compared with 13 (10.4%) in the intervention group (*P*=.26). The Kaplan-Meier estimated median time for the first 15% of the cohort to return to work was 87.1 weeks (95% CI 60.0-109.1 weeks) for the matched control group compared with 70.6 weeks (95% CI 52.6-79.6 weeks; *P*=.08) for the intervention group.

**Conclusions:**

Patients receiving a remotely delivered coaching program in a real-world setting returned to work at a higher frequency than did control participants receiving usual care.

## Introduction

Early detection and sustained improvements in the treatment of many types of cancer have markedly improved survivorship rates [1]. Approximately 45% of cancer diagnoses occur in people of working age, between 20 and 64 years old [2,3], and it is likely that the prevalence of cancer survivorship in the workforce will continue to increase.

For working age cancer survivors, impairments in physical and mental health from the disease sequelae or side effects of treatment may reduce their participation in work [4,5]. Specifically, cancer survivors are at higher risk of unemployment [4,6,7], reduced hours, prolonged absenteeism [8,9], and impaired presenteeism [9] compared with individuals without a history of cancer. Returning to work is important for cancer survivors themselves, their employers, and the society at large [4,8,9]. For cancer survivors, returning to work can improve their sense of “normality,” their self-respect [10], and their quality of life [11,12]. Conversely, prolonged job loss increases the risk of financial toxicity, resulting from decreased earnings and increased health expenditure. Financial toxicity following a cancer diagnosis is associated with emotional distress, poor treatment adherence, and a higher mortality risk [8]. From an employer and societal perspective, the return to work of knowledgeable and experienced workers enables continuity of a skilled labor pool, along with reduced productivity losses and decreased expenses like disability claim payments [13].

Factors that have been identified to adversely influence return to work include cancer diagnosis, including head and neck [5,7], central nervous system, and advanced blood and lymph malignancies [4,5]; type of work, particularly manual labor [7,10]; treatment, especially certain surgeries and systemic therapy [4,5,8]; lacking a supportive environment, including work flexibility [7,10], financial situation, and insurance availability and type [12]; and greater physical limitations [5,6]. Age and other demographics have historically had mixed influences [7], although more recently favoring successful return to work of younger employees [6] and those with higher education levels [6,8].

Return to work has therefore become a pressing issue and key unmet need of this population. A previous meta-analysis of 5 multidisciplinary interventions that covered physical, psychoeducation, and/or vocational components showed moderate evidence for improving return to work rates [14]. These interventions were provided from hospital settings to narrowly defined populations and delivered in-person, which can be both time intensive and costly. Among 3 interventions identified in a systematic review for return-to-work interventions outside of the hospital setting [15], only 1 had a suitable comparison group, but with no demonstrated effect [16]. Therefore, a paucity of evidence exists for multidisciplinary interventions provided as routine employment support that serve broad populations and adapt to the complexities and diversity of day-to-day cancer care and life in general.

In 2018, a multidisciplinary intervention delivered via digital resources and calls with a health coach was introduced by AIA Australia, a life and health insurance company, to its members with a disability insurance policy claim. This study sought to evaluate the long-term impact of the program on return-to-work outcomes as compared to usual care.

## Methods

### Study Design

The study is a propensity score–matched retrospective cohort analysis. Eligible AIA members were enrolled in the intervention, the CancerAid Coach Program, from October 2018 to February 2020. A comparison group was created using the below criteria and then abstracted from deidentified records of patients who did not participate in the intervention (see Multimedia Appendix 1 for a visual representation of the trial design and median times). The CancerAid online eHealth app is freely available for cancer survivors and carers for iOS [17] and Android [18].

### Recruitment and Eligibility Criteria

From October 2018 to February 2020, during routine calls following lodgment of a disability claim for a cancer diagnosis, AIA staff had private conversations with potential participants to elicit their interest in participating in the intervention. Eligibility for a disability claim included patients who (1) were of working age (18-65 years); (2) held a disability insurance policy through their insurer (AIA Australia) that included coverage of a cancer diagnosis; and (3) were working prior to diagnosis and were unable to work in their regular prediagnosis capacity for at least 3 months. Program enrollment involved the AIA staff member eliciting interest and completing a secure web form, followed by automated email outreach that included consent for the use of deidentified data for research purposes [19]. The inclusion criteria were as follows: (1) completing enrollment and having at least one or more calls with a health coach; (2) a minimum follow-up time from diagnosis of 34 weeks to allow for completion of the intervention (median 10 weeks) along with delays in lodgment of the claim with the insurer (median 12 weeks) and a subsequent delay in referral to the CancerAid Coach Program (median 12 weeks); and (3) diagnosis from the top 10 most common cancer types (breast, including in situ and invasive, brain, lung, colon, ovary, pancreas, prostate, and lymphoma malignancies) to enable adequate matching. The exclusion criteria were patients whose policies were later withdrawn or who did not meet the eligibility criteria of their disability insurance policy.

### Intervention and Usual Care

The CancerAid Coach Program provides a range of integrative therapies to help manage symptoms and adverse effects during or after treatment. The CancerAid Coach Program is based upon lifestyle and psychological interventions that are well established and consistent with American Society of Clinical Oncology (ASCO) guidelines (eg, diet and exercise in survivors of cancer, and peer support) [20,21] or backed by evidence from large randomized trials to improve patient outcomes (eg, digital symptom tracking) [22]. By focusing on interventions demonstrated to improve patient outcomes, it is predicted that return-to-work outcomes will also increase as patients now encounter fewer impairments in physical and mental health [5].

The CancerAid Coach Program consists of an online eHealth app (see Multimedia Appendices 2 and 3) and 3 telephone health coaching sessions delivered over a 12-week period. Additionally, a series of weekly messages, via email and text, are sent to participants during the period of the intervention to help reinforce key health messages on appropriate symptom tracking, exercise, diet, mindfulness, and sleep strategies. The CancerAid app allows patients to coordinate their care with tools to read about their condition, treatment options, and a broader community of cancer survivors. It also allows patients to monitor their condition, specifically in relation to being able to track their symptoms digitally and monitor their diet, exercise, sleep, and other patient-level data at home via the app.

The health coach team includes registered nurses, doctors, and allied health professionals. Coaches offer a range of interventions tailored to the needs and current stage of each patient, and use principles of behavioral change theories, such as the transtheoretical model of stages of change [23]. These interventions include inviting patients to consider their current behavior; helping them consider the impacts of making change; providing encouragement, support, and feedback on performance; encouraging patients to set further goals once existing goals are met; and finally, providing a framework of accountability. The eHealth app and regular text and email messages reinforce these interventions and help overcome many barriers to seeking face-to-face support. These interventions are applied to each of the key health messages to help improve the uptake of frequent symptom tracking, appropriate exercise and dietary intake, mindfulness, and sleep hygiene strategies.

Usual care consisted of regular phone calls with AIA staff members, for example, every few weeks, along with an optional referral to 2 rehabilitation programs consisting of support with an exercise physiologist or an occupational rehabilitation consultant. Participation in these 2 rehabilitation programs were at the patient’s discretion, and participating in the CancerAid Coach Program did not preclude participation in either of these 2 rehabilitation programs.

### Matched Comparison Group

The intervention group of Coach Program participants were matched on a one-to-one basis to a control group of nonparticipating insurance plan members who were otherwise eligible to participate using propensity score matching. Controls were first collected from the AIA claims database over the same period (October 2018 to February 2020) and using the same inclusion criteria as follows: (1) working age; (2) disability claim for a cancer diagnosis; (3) inability to work in their regular capacity for at least 3 months; (4) minimum follow-up time from diagnosis of 34 weeks; and (5) top 10 most common cancer types.

A logit regression model was used to calculate a propensity score for each participant, to represent the probability that they would be referred to the CancerAid group. The covariates of the propensity model included age, gender, insurance benefit type, date of cancer diagnosis, and time from diagnosis to lodgment of the claim. Using the propensity scores, CancerAid participants were matched on a one-to-one basis with the nearest-neighbor method without replacement to create a matched control group. The baseline characteristics were then reassessed for imbalance using absolute standardized mean difference.

### Assessment and Outcomes

Outcome measures were derived from insurance claims data as standard business practice. Primary outcomes were rates of (1) returning to work; (2) death; and (3) claim closure, other than death or returning to work. The durations of returning to work and claim closure, commencing from the date of a cancer diagnosis, were also reported. The reasons for claim closure, other than death or returning to work, included a single lump-sum payment (compared to scheduled salary replacement), expiry of the benefit period (meaning the insurance policy had expired as set out in the policy’s schedule), no longer meeting the definition of disability (ie, return to health but not work), and abandonment of the claim. A claim reported as open meant none of the previously mentioned outcomes had occurred.

### Statistical Analysis

Statistical analysis was performed in R (version 4.0.3; R Foundation for Statistical Computing). Variables for propensity score matching included age, gender, cancer diagnosis, date of cancer diagnosis, time to lodgment, insurance benefit type, occupation, and geography setting. Geography settings were defined by the Australian Government as follows: 1, major cities; 2, cities and major regional centers; and 3, regional centers and other regional areas [24]. The difference in the final return to work rate was tested using a chi-squared test without Yates correction (significance *P*<.05). The time from diagnosis to return-to-work claim closure was calculated using a Kaplan-Meier model evaluated with a log-rank test (significance *P*<.05).

## Results

### Overview

A total of 220 participants were enrolled in the intervention, of which 125 met the criteria for this analysis (see Multimedia Appendix 4 for patient flow). A further 3749 participants who did not receive the intervention over the same period were identified from the insurer’s records. Of these, 1856 control group participants met the criteria for analysis. There were observed imbalances in baseline characteristics between the intervention and control cohorts, including sex, tumor origin, geography setting, and benefit period. Based on 1:1 matching with nearest-neighbor matching, 125 intervention patients were matched to 125 control patients.

### Propensity Score Results

After matching using the propensity scores, 125 intervention patients were matched to 125 control patients. The C-statistic for the logistic regression was 0.66. Covariates in the propensity match were overall well balanced, with an absolute standardized difference less than 0.1 for age, date of diagnosis, time to lodge a claim from diagnosis, and benefit period in the matched groups. The absolute standardized difference was 0.11 for gender, with 94.4% (118/125) of intervention participants being female versus 91.2% (114/125) of control participants (*P=*.32).

The occupational category and the tumor origin site were balanced between the matched groups (Table 1). There was a difference in the geographical setting between groups, with 42.4% (53/125) of intervention participants being from major cities versus 28.8% (36/125) of control participants (*P*=.03). However, a separate analysis revealed there was no correlation between geographical setting and any of the primary outcomes, including return to work (*P*=.43), for the control and intervention groups. Geographical setting could not be addressed in propensity score matching as it does not lead to acceptable standardized mean differences between groups. Other clinical and demographic variables (age, gender, tumor origin, rehabilitation referral, and occupational category) were not statistically different between groups.

**Table 1:** Baseline demographic and clinical characteristics.

Characteristic		All participants				Propensity score–matched participants				Standardized mean difference
		Control (n=1856)	Intervention (n=125)	*P* value	Control (n=125)		Intervention (n=125)	*P* value		
**Age (years)**				.96				.56	0.03	
	Median	52	53		53		53			
	IQR	45-59	45-58		47-59		45-58			
**Sex, n (%)**				<.01				.33	0.11	
	Female	1513 (81.5)	114 (91.2)		118 (94.0)		114 (91.2)			
	Male	343 (18.5)	11 (8.8)		7 (5.6)		11 (8.8)			
**Geographical setting, n (%)**				.04				.03	N/A^a^	
	1: major cities	663 (35.7)	53 (42.4)		36 (28.8)		53 (42.4)			
	2: cities and major regional centers	668 (36.0)	31 (24.8)		48 (38.4)		31 (24.8)			
	3: regional centers and areas	525 (28.3)	41 (32.8)		41 (32.8)		41 (32.8)			
**Tumor origin, n (%)**				.03				.27	N/A	
	Breast	911 (49.1)	76 (60.8)		66 (52.8)		76 (60.8)			
	Brain	147 (7.9)	6 (4.8)		9 (7.2)		6 (4.8)			
	Colon	258 (13.9)	17 (13.6)		15 (12)		17 (13.6)			
	Hodgkin lymphoma	68 (3.7)	6 (4.8)		4 (3.2)		6 (4.8)			
	Non-Hodgkin lymphoma	60 (3.2)	5 (4.0)		7 (5.6)		5 (4.0)			
	Ovary	90 (4.8)	7 (5.6)		7 (5.6)		7 (5.6)			
	Pancreas	71 (3.8)	5 (4.0)		4 (3.2)		5 (4.0)			
	Prostate	59 (3.2)	0 (0.0)		0 (0.0)		0 (0.0)			
**Rehabilitation referral, n (%)**				.13				.70	N/A	
	No	1165 (62.8)	70 (56.0)		67 (53.6)		70 (56.0)			
	Yes	691 (37.2)	55 (44.0)		58 (46.4)		55 (44.0)			
**Benefit period, n (%)**				.03				.70	0.01	
	1 year	7 (0.4)	0 (0.0)		1 (0.8)		0 (0.0)			
	2 years	523 (26.4)	23 (18.4)		25 (20.0)		23 (18.4)			
	5 years	120 (6.1)	7 (5.6)		4 (3.2)		7 (5.6)			
	Age 60 years	616 (31.1)	51 (40.8)		43 (34.4)		51 (40.8)			
	Age 65 years	216 (10.9)	7 (5.6)		9 (7.2)		7 (5.6)			
	Age 67 years	486 (24.5)	35 (28.0)		42 (33.6)		35 (28.0)			
	Age 70 years	3 (0.2)	0 (0.0)		1 (0.8)		0 (0.0)			
**Occupation category, n (%)**				.21				.37	N/A	
	Armed forces occupations	2 (0.1)	0 (0.0)		0 (0.0)		0 (0.0)			
	Clerical support worker	206 (11.1)	16 (12.8)		16 (12.8)		16 (12.8)			
	Craft and related trade worker	57 (3.1)	3 (2.4)		3 (2.4)		3 (2.4)			
	Elementary occupations	88 (4.7)	3 (2.4)		4 (3.2)		3 (2.4)			
	Manager	194 (10.5)	10 (8.0)		11 (8.8)		10 (8.0)			
	Plant and machine operator, and assembler	31 (1.7)	3 (2.4)		1 (0.8)		3 (2.4)			
	Professional	416 (22.4)	21 (16.8)		36 (28.8)		21 (16.8)			
	Service and sales worker	592 (31.9)	50 (40.0)		39 (31.2)		50 (40.0)			
	Skilled agricultural, forestry, and fishery worker	6 (0.3)	2 (1.6)		0 (0.0)		2 (1.6)			
	Technician and associate professional	243 (13.1)	17 (13.6)		14 (11.2)		17 (13.6)			
	Unknown	21 (1.1)	0 (0.0)		1 (0.8)		0 (0.0)			
**Time to lodge a claim (weeks)**				<.01				.48	0.03	
	Average	27.8	17.8		18.6		17.8			

^a^N/A: not applicable.

### Outcomes

**Figure 1:**
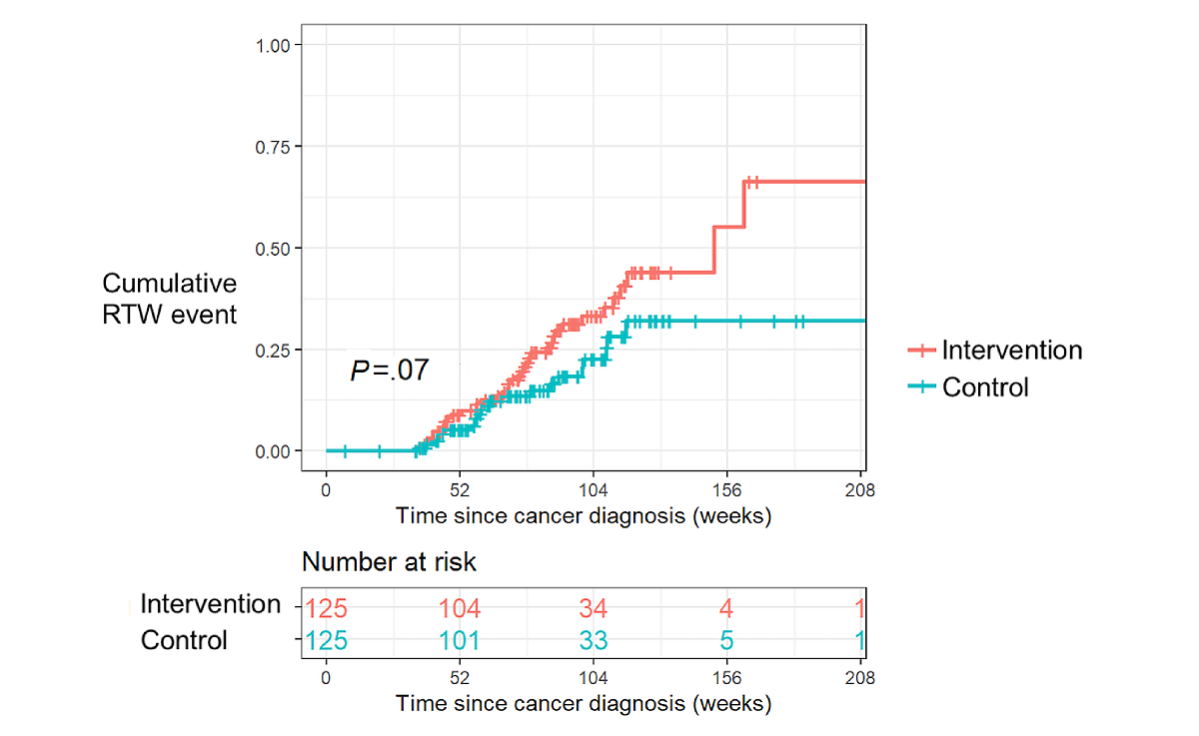
Cumulative event plot of returning to work (intervention vs matched control group). RTW: return to work.

Outcomes are listed in Table 2 and illustrated in Figure 1. The median follow-up since cancer diagnosis was 79 weeks (IQR 60-106 weeks). In the matched control group, 22 (17.6%) participants returned to work compared with 38 (30.4%) in the intervention group (*P*=.02). Additionally, 19 (15.2%) matched control participants died prior to claim closure compared with 13 (10.4%) in the intervention group (*P*=.26). When considering survivorship only, the return to work rate was 33.9% (38/112) in the intervention group compared with 20.8% (22/106) in the matched control group (*P*=.03). No difference was identified between the control and intervention groups for the duration or rate of claim closure arising from causes other than return to work or death (Table 2). Expiry of the benefit period and abandonment of a disability claim were the most cited reasons for claim closure in both the control and intervention groups (Table 3).

**Table 2:** Outcomes.

Characteristic			Propensity score–matched participants				*P* value
			Control (n=125)		Intervention (n=125)		
**Return to work**							
	Value, n (%)	22 (17.6)		38 (30.4)		.02	
	Duration (weeks), median	60		71		.62	
	Duration (weeks), IQR	49-88		49-94			
**Claim closure (no return to work or death)**							
	Value, n (%)	20 (16.0)		16 (12.8)		.12	
	Duration (weeks), median	68		71		.62	
	Duration (weeks), IQR	55-99		49-94			
**Claims open**							
	Value, n (%)	64 (51.2)		58 (46.4)		.71	
**Death**							
	Value, n (%)	19 (15.2)		13 (10.4)		.26	

**Table 3:** Claim closure outcomes (other than return to work or death).

Claim closure reason	Propensity score–matched participants	
	Control (n=125), n	Intervention (n=125), n
Abandoned	8	5
No longer meets the definition of disability	5	1
Expiry of the benefit period	7	9
Lump sum paid	0	1

The cumulative event plot of returning to work for the matched participants is presented in Figure 1. Further analysis showed that the estimated return to work rate at 2 years after a cancer diagnosis was 33.1% (95% CI 22.4-42.3%) for the intervention group compared with 22.6% (95% CI 12.3-31.8%) for the control group. The median time for the first 15% of the cohorts to return to work was 70.6 weeks (95% CI 52.6-79.6 weeks) for the intervention group compared with 87.1 weeks (95% CI 60.0-109.1 weeks) for the matched control group.

## Discussion

This study evaluated the impact of a remotely delivered coaching program combined with digital support for patients diagnosed with cancer. An increase of 12.8% in the return to work rate was identified for coach program participants over an 18-month period compared with matched controls. These results are consistent with clinical-based trials of in-person multidisciplinary interventions that have been shown to enhance return to work [14]. Furthermore, this study demonstrates that support programs can be effectively implemented as part of routine employment support and remotely delivered outside of the hospital setting. The median time to return to work showed a nonsignificant trend favoring coach program participants versus matched controls. A maturing data set and greater study numbers may in time reveal the true effect (or not) of the program intervention on median time.

The return to work rates identified in this study are comparable to those in existing literature when factoring a baseline minimum of 3 months of absence from work and a definition of returning to a prediagnosis work capacity at 1.5 years (33.9% for the intervention group and 20.8% for the matched control group). For example, large cohort studies have shown that approximately 60% of cancer survivors successfully return to work at 1 to 2 years after a cancer diagnosis, but noted that the majority will have reduced hours either permanently or over a time limited period [6,7,25-27]. Another important difference between this study and cohort studies that may underestimate the true rate of returning to work among cancer survivors is that some individuals diagnosed with cancer may remain employed or have adequate leave (eg, sick leave and annual leave) that avoids the need for a claim on their disability insurance policy. Finally, this study precluded those with an early claim closure (less than 34 weeks), to allow for a suitable referral period for the intervention, which would similarly underestimate the true rate of returning to work among working-age cancer survivors.

Returning cancer survivors to the workplace mitigates against financial toxicity for the individual, while reducing the economic burden of cancer on payers and employers [28]. Other studies have demonstrated the cost-effectiveness of coaching interventions delivered remotely and for the routine support of employees diagnosed with chronic diseases, such as diabetes, cardiovascular diseases, and respiratory diseases [29-34]. Notably, cost savings have not been demonstrated with low-intensity coaching (average of 2 calls each) and delivered over 12 months or less [31-33]. For this study, no difference between the intervention and matched controls for returning to work was observed within 12 months of diagnosis (Figure 1). Possible explanations for this include a delay in receiving the intervention, with an average period of 24 weeks between a cancer diagnosis and first receiving the intervention. Additionally, for the present intervention to be successful, the barriers to returning to work must be amenable to the adoption of healthy behaviors and self-management principles. Many patients receiving active cancer treatment have associated toxicities that are known to impair short-term work ability [6,35], and these may not be immediately amenable to improvements in self-management. The results of this study complement other recent studies showing the receptiveness of cancer survivors to digital technology for the support of physical rehabilitation [36,37], along with demonstrated improvements in quality of life through digital support [38].

Employment after a cancer diagnosis is an important social determinant of health [3] and is associated with improved quality of life and the magnitude of the cancer health burden [11,12,39]. Hence, coaching support that is implemented as part of routine care and made accessible to broader populations will typically provide reductions in medical expenditure. Additional cancer rehabilitation that would advance the current intervention while improving function in survivors and decreasing the economic burden of cancer for individuals and the society includes rehabilitation for pain, musculoskeletal issues, deconditioning, balance, and lymphedema [28].

This study has several limitations. Individuals were not randomized to participate; hence, there may be differences in motivation for opting to participate in the program compared with the matched control group, and this could not be balanced out through propensity score matching. Other researchers have shown that the wish to participate in support programs is usually an indicator of the need for greater assistance with health and knowledge [40]. Conversely, high motivation for opting to participate may overestimate the program’s effect when applied to a generalized setting. Socioeconomic status, which may substantially differ between coaching participants and controls, was not available for use in the propensity score models. However, program participants and matched controls had comparable rates of occupation. Similarly, propensity scores were used for benefit type, as an indication of the level of insurance and by proxy the level of income, and both factors would likely address socioeconomic status. The type of treatment and stage of disease, both known factors of return-to-work outcomes, were not captured in this insurance data set and therefore were not available for matching. Finally, the overrepresentation of females, likely a result of opting in, and certain occupations, and the inclusion of the top 10 cancer types could somewhat reduce the generalizability of the results.

The study findings indicate that patients diagnosed with cancer and receiving a remotely delivered coaching program in a real-world setting returned to work at a higher frequency than did control participants receiving usual care. The results of this study add to the literature of cancer as a chronic and manageable disease in the workplace.

## Appendix

Multimedia Appendix 1 Trial design and median times. RTW: return to work.

Multimedia Appendix 2 Screenshot of the CancerAid smartphone application.

Multimedia Appendix 3 Screenshot of the CancerAid smartphone application.

Multimedia Appendix 4 Patient flow.
