# Supplementary figures and images for: A Digital Coaching Intervention for Cancer Survivors With Job Loss: Retrospective Study
Source: JMIR Cancer. 2021 Nov 23;7(4):e31966. doi: 10.2196/31966 (PMC8663674; doi:10.2196/31966)

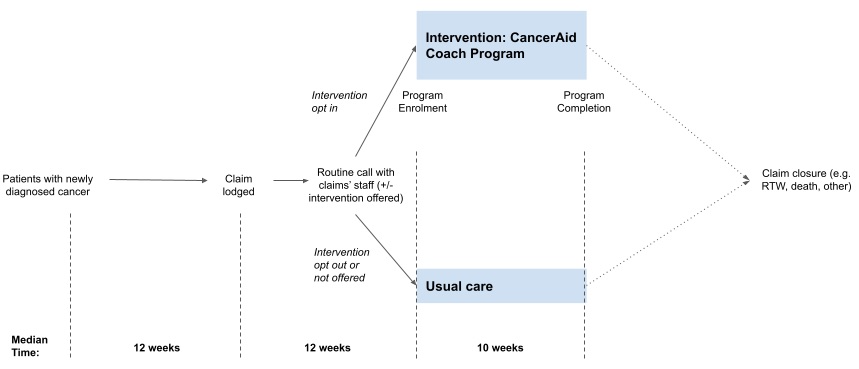

Supplement: Multimedia Appendix 1 [file cancer_v7i4e31966_app1.png]

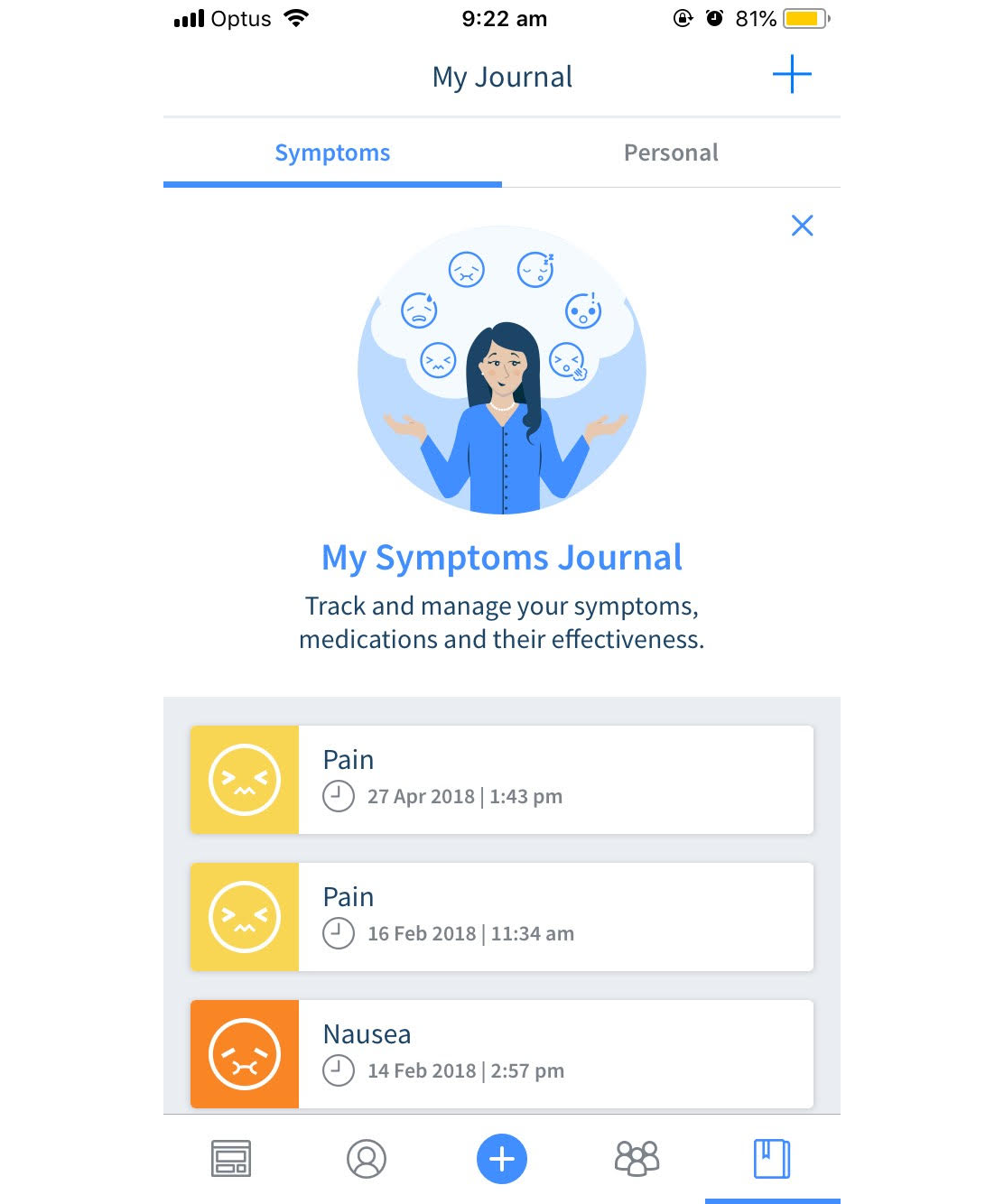

Supplement: Multimedia Appendix 2 [file cancer_v7i4e31966_app2.png]

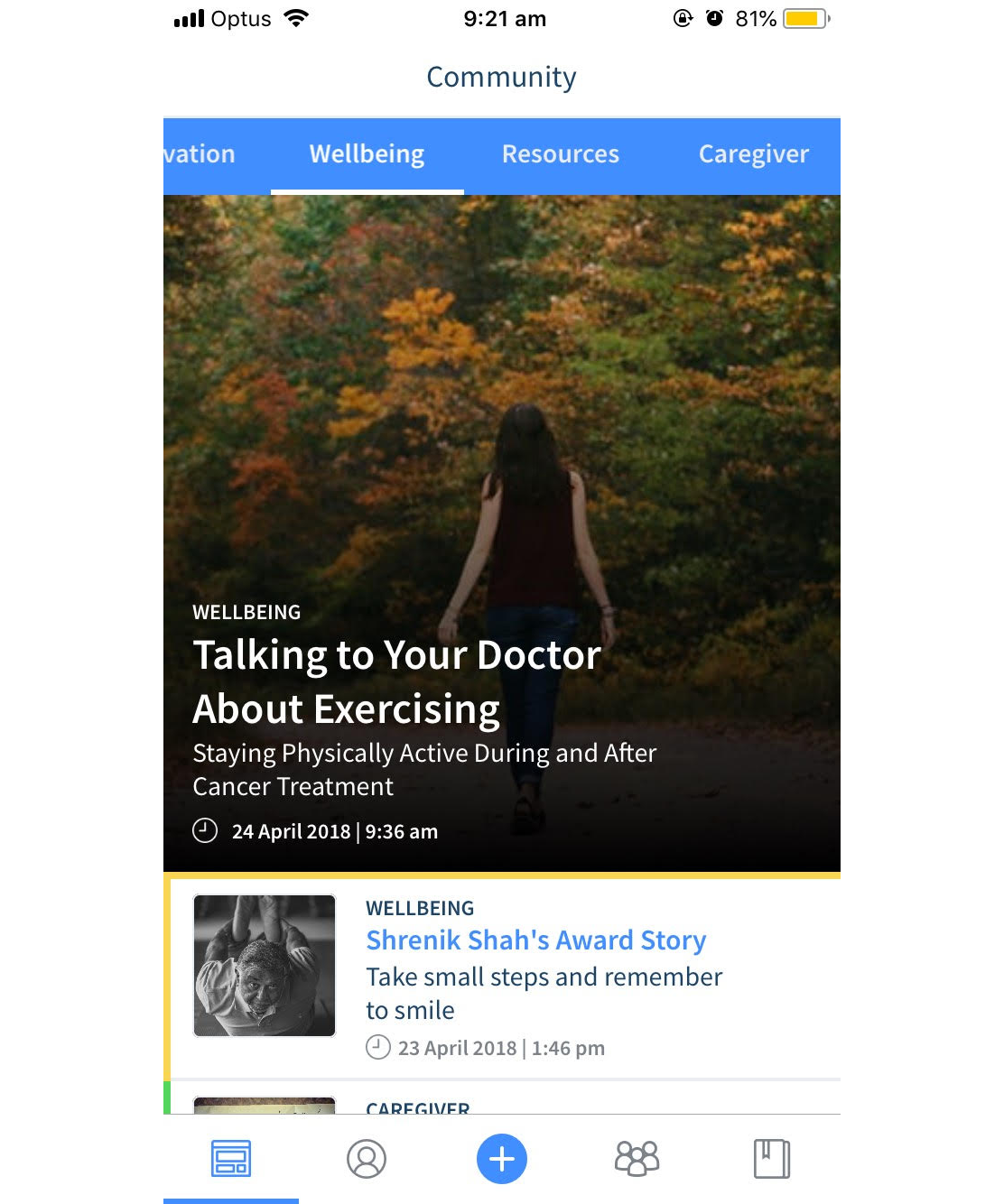

Supplement: Multimedia Appendix 3 [file cancer_v7i4e31966_app3.png]

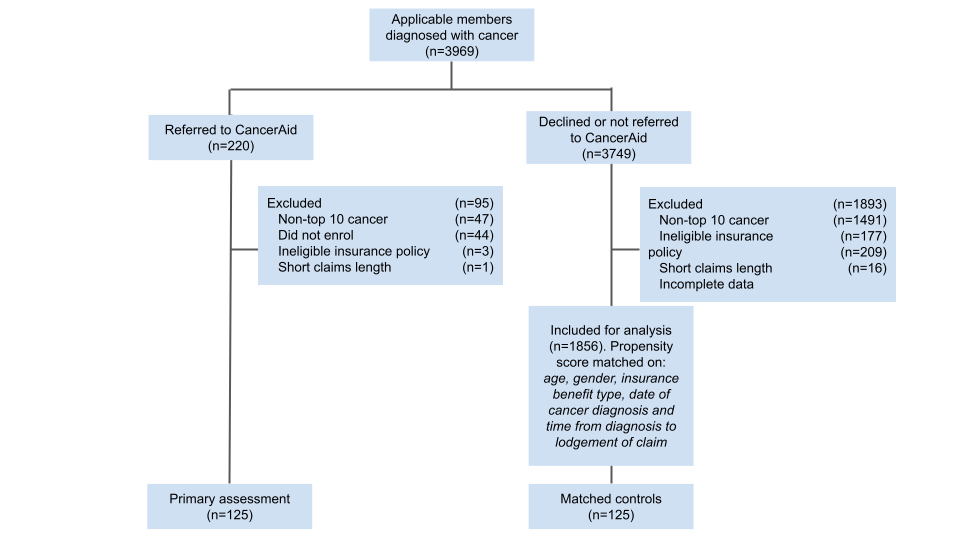

Supplement: Multimedia Appendix 4 [file cancer_v7i4e31966_app4.png]
